# Lactate index and survival in hospital-acquired septic shock

**DOI:** 10.1186/cc9463

**Published:** 2011-03-11

**Authors:** S Omar, Mathivha, J Dulhunty, A Lundgren

**Affiliations:** 1 University of Witwatersrand, Johannesburg, South Africa; 2 University of Queensland, Brisbane, Australia

## Introduction

Severe sepsis is characterised by profound metabolic and inflammatory derangement, which can lead to multiorgan failure and death. During septic shock, oxygen delivery may fail to meet tissue demand resulting in increased oxygen extraction. Once tissue needs are no longer met, an oxygen debt with global tissue hypoxia and associated hyperlactataemia ensues. Several studies have shown that blood lactate may be used as a marker of global tissue hypoxia and prognosis in shock states.

## Methods

Forty patients requiring adrenaline therapy for a first episode of septic shock acquired >24 hours after admission to the ICU had blood lactate levels measured 2-hourly over a 24-hour period. Adrenaline therapy was escalated until the target mean arterial pressure was reached. The lactate index was calculated as the ratio of maximum lactate increase to the adrenaline increase.

## Results

Lactate increased from 2.3 to 2.9 mmol/l (*P *= 0.024) and the mean adrenaline increase was 0.14 μg/kg/minute. Peak lactate correlated with peak adrenaline (rho = 0.34, *P *= 0.032). Lactate index was the only independent predictor of survival after controlling for age and APACHE II score (OR = 1.14, 95% CI = 1.03 to 1.26, *P *= 0.009).

## Conclusions

A high lactate following adrenaline administration may be a beneficial and appropriate response.
